# An Updated Review of Topical Tretinoin in Dermatology: From Acne and Photoaging to Skin Cancer

**DOI:** 10.3390/jcm14227958

**Published:** 2025-11-10

**Authors:** Pablo Balado-Simó, Daniel Morgado-Carrasco, Sara Gómez-Armayones, Anna López-Ferrer, Didac Barco, Carla Ferrándiz-Pulido, Sebastian Podlipnik

**Affiliations:** 1 Dermatology Department, Hospital Clínic de Barcelona, Universitat de Barcelona, 08007 Barcelona, Spain; 2 Dermatology Department, Hospital de Figueras, Fundació Salut Empordà, 17600 Figueras, Spain; 3 Clínica Corium Dermatology, 08007 Barcelona, Spain; 4 Dermatology Department, Hospital de la Santa Creu i Sant Pau, 08041 Barcelona, Spain; 5 Dermatology Department, Hospital Universitari Vall d’Hebron, 08035 Barcelona, Spain; 6 Institut d’Investigacions Biomèdiques August Pi i Sunyer (IDIBAPS), 08036 Barcelona, Spain

**Keywords:** tretinoin, dermatology, acne, photoaging, skin cancer, treatment

## Abstract

Topical tretinoin (all-trans-retinoic acid) is a first-generation vitamin A derivative with well-established efficacy in acne vulgaris and photoaging. Owing to its pleiotropic effects on epidermal differentiation, collagen synthesis, and skin pigmentation, numerous off-label uses have been proposed across dermatology. This narrative review summarizes current evidence on the efficacy and safety of topical tretinoin for multiple dermatological conditions, based on studies published between January 2000 and July 2025. Robust data from randomized clinical trials (RCTs) and systematic reviews support its benefit in acne and photoaging, whereas smaller RCTs and prospective studies indicate potential efficacy for melasma, postinflammatory hyperpigmentation, striae distensae, flat warts, alopecia areata, androgenetic alopecia, hypertrophic scars and keloids, and actinic keratosis and as pretreatment before chemical peels or laser resurfacing. However, high-quality, adequately powered trials with standardized outcome measures are still needed to establish clinical guidelines. Regarding cutaneous oncology, a large RCT demonstrated no preventive effect of tretinoin on keratinocyte carcinomas. Adverse events are typically mild, localized, and transient, and available evidence does not support an association with systemic adverse effects.

## 1. Introduction

**Figure 1:**
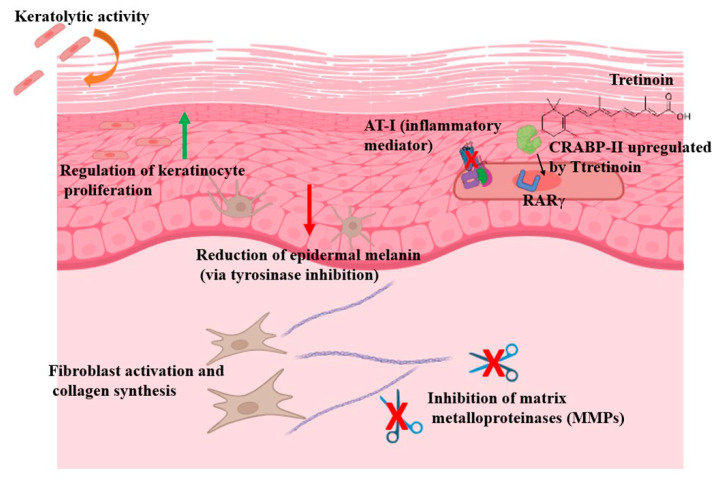
Effects of topical tretinoin on skin. Image created with BioRender.com.

Topical tretinoin (all-trans-retinoic acid) is a potent first-generation vitamin A derivative with a complex underlying mechanism of action (Table 1, Figure 1). This includes keratolytic activity, regulation of proliferation and differentiation of epidermal cells, activation of fibroblasts, induction of collagen synthesis and collagen recycling, prevention of collagen loss, reduction in matrix metalloproteinases (MMPs) 1 and 8, and a decrease in epidermal melanin through inhibition of tyrosine kinase [1]. Tretinoin can activate nuclear retinoic acid receptors (RARα, RARβ, and RARγ), which subsequently mediate gene expression, particularly via RARβ and RARγ. These receptor interactions play a crucial role in blocking inflammatory mediators and in normalizing skin cell growth [2]. This multifaceted mechanism, encompassing cellular proliferation and differentiation, inflammation modulation and extracellular matrix remodeling, explains the effectiveness of tretinoin in diverse dermatological disorders [3]. The US Food and Drug Administration (FDA) approved topical tretinoin for acne vulgaris in 1971 and for the palliative treatment of photoaging in 1995. However, it has been widely used off-label in dermatology, including for the management of scarring [4], infections [5], inflammatory dermatoses [6] and pigmentary disorders [7], and the prevention and treatment of skin cancer [8].

Here, we review the usefulness and safety of topical tretinoin for the treatment of diverse dermatological disorders, photoaging, and skin cancer.

## 2. Methods

The objective of this narrative review was to identify, summarize, and critically evaluate clinical evidence supporting approved and off-label uses of topical tretinoin in dermatology. We conducted the review following the recommendations of SANRA (*Scale for the Assessment of Narrative Review Articles*) [9]. The search was performed in PubMed/MEDLINE and Google Scholar, covering articles published from 1 January 2000 to 31 July 2025. The following search terms and their combinations were used: “*tretinoin*”, “*all-trans retinoic acid*”, “*treatment*”, “*dermatology*”, “*acne*”, “*rosacea*”, “*flat wart*”, “*photoaging*”, “*melasma*”, “*pigmentary disorders*”, “*acanthosis nigricans*”, “*striae distensae*”, “*scars*”, “*keratinization disorders*”, “*psoriasis*”, “*dermatoses*”, “*skin cancer*”, “*actinic keratosis*”, “*energy-based*”, “*laser*”, “*botulinum toxin*”, “*alopecia*”.

No geographic restrictions were applied. Only full-text peer-reviewed articles were considered. We included articles published in English or Spanish that met the following criteria:

- Primary inclusion: Randomized controlled trials (RCTs) and prospective clinical studies.
- Secondary inclusion: Retrospective studies with >20 patients when prospective data were unavailable.
- Exclusion: Case reports, case series ≤20 patients, animal/in vitro studies, conference abstracts without full text, and studies on systemic retinoids only.

Titles and abstracts were independently screened by two reviewers (PBS and DMC). Full texts meeting inclusion criteria were retrieved and disagreements were resolved by reaching consensus. For each included study, we extracted study design, population, sample size, intervention details (formulation, concentration, regimen), comparator(s), main efficacy outcomes, follow-up duration and reported adverse events. Given the heterogeneity of study designs and outcome measures, data were synthesized narratively and, where possible, summarized in comparative tables.

## 3. Results: Dermatological Applications of Topical Tretinoin

### 3.1. FDA-Approved Indications

#### 3.1.1. Acne Vulgaris (Table 2)

The efficacy and safety of topical tretinoin in acne vulgaris have been extensively evaluated in multiple randomized clinical trials (RCTs) across various age groups and formulations. A multicenter RCT (n = 178) showed that tretinoin 0.04% gel significantly reduced inflammatory and non-inflammatory lesions at week 12 compared to the vehicle employed (*p* < 0.05), with good overall tolerability [10]. In a separate comparative trial (n = 156), tretinoin 0.04% and 0.1% gels showed similar efficacy, while the lower concentration exhibited a more favorable safety profile [11].

**Table 2:** Main studies on tretinoin as treatment for acne vulgaris.

Study	Design	Population	Intervention	Duration	Main Outcomes
Berger et al. [10], 2007	Double-blind, multicenter RCT	178 subjects (≥12 years) with acne vulgaris	Tretinoin microsphere 0.04% vs. vehicle	12 weeks	Significant reduction in total inflammatory and non-inflammatory lesions (35.5%, 38.2%, and 33.6% vs. 20.9%, 19.2%, and 20.4%; all *p* < 0.05).
Berger et al. [11], 2007	Randomized comparative study	156 subjects with mild-to-moderate acne	Tretinoin microsphere 0.04% vs. tretinoin 0.1%	12 weeks	Both effective; tretinoin 0.04% showed better tolerability.
Cook-Bolden et al. [12], 2019	Post hoc analysis of RCTs	766 Hispanic subjects (11–50 years) with moderate-to-severe acne	Tretinoin 0.05% lotion vs. vehicle	12 weeks	Reduction in inflammatory (60.1% vs. 51.1%) and non-inflammatory (53% vs. 38.7%) lesions; treatment success (≥2-grade improvement in EGSS) in 19.6% vs. 12.7% with vehicle (*p* = 0.015).
Dogra et al. [13], 2021	Randomized, comparative study	750 patients with mild-to-moderate acne	Tretinoin 0.04% + clindamycin 1% vs. clindamycin 1% vs. tretinoin 0.04%	12 weeks	Mean reduction in lesion count: inflammatory, −77%; non-inflammatory, −71%; total lesions, −73%. Proportion of patients achieving ≥2-grade ISGA improvement and rated as “clear” or “almost clear”: 46% with combination vs. 31% with tretinoin monotherapy and 33% with clindamycin monotherapy (*p* < 0.02).
Draelos et al. [14], 2012	Randomized, double-blind study	66 subjects (≥18 years) with moderate acne	BPO 5.5% + tretinoin 0.025% vs. clindamycin 1% + BPO 5% + tretinoin 0.025%	12 weeks	Both regimens showed significant reductions in inflammatory and non-inflammatory lesions, with comparable tolerability. Mean reduction in total lesion count: 66.7% vs. 73.9%. Statistically significant improvement from baseline in skin tone, brightness, pores, and overall appearance. Mild, transient irritation observed in both groups resolved by week 8.
Eichenfield et al. [15], 2008	Phase IV, open-label trial	544 subjects (≥12 years) with mild-to-moderate acne	Tretinoin gel microsphere (TGM) 0.04% (n = 361) or 0.1% (n = 183), applied once daily via pump	12 weeks	Significant improvement in mGAGS score from baseline to week 12: −4.2 (0.04%) and −4.7 (0.1%) (both *p* < 0.0001). At week 12, 72% of patients showed ≥ moderate improvement (IGE), and 25% were rated as clear or almost clear. High treatment compliance (≈95%) and low rate of adverse events (13.6%, mostly mild). Both concentrations well tolerated and effective.
Eichenfield et al. [16], 2012	Randomized, double-blind trial	110 preadolescents (9–11 years) with acne vulgaris	Tretinoin microsphere 0.04% pump vs. vehicle	12 weeks	Significant reduction in non-inflammatory lesions with tretinoin vs. vehicle (–19.9 vs. −9.7; *p* = 0.04). IGA improvement (≥2-point) achieved in 25.5% with tretinoin vs. 13.0% with vehicle (*p* = 0.02). No significant differences in static IGA or inflammatory lesions. Cutaneous irritation was mild and comparable between groups.
Han et al. [17], 2019	Post hoc analysis of RCTs	69 Asian patients (12–48 years, 61% female) with moderate-to-severe acne	Tretinoin 0.05% lotion vs. vehicle	12 weeks	Reduction in inflammatory (58.6% vs. 41.5%) and non-inflammatory (51.4% vs. 23.9%) lesions (*p* = 0.012 for non-inflammatory lesions); QoL improved; well tolerated.
Harper et al. [18], 2019	Post hoc analysis of RCTs	606 adult females (≥18 years) with moderate-to-severe acne (EGSS 3–4)	Tretinoin 0.05% lotion vs. vehicle, once daily	12 weeks	In moderate acne, tretinoin achieved greater lesion reductions (inflammatory 58.5% vs. 50.3%; non-inflammatory 55.5% vs. 39.8%; both *p* < 0.05) and higher treatment success (25.4% vs. 15.4%; *p* = 0.006). QoL and patient satisfaction significantly improved. In severe acne, lesion reductions were numerically greater but not significant. Most AEs were mild; application site pain (2.9%) and dryness (5.0%) were most frequent.
Tyring et al. [19], 2018	Double-blind RCT	1640 patients with moderate-to-severe acne	Tretinoin 0.05% lotion vs. vehicle	12 weeks	Reduction in inflammatory (52%) and non-inflammatory (46%) lesions; 18% treatment success; favorable safety profile.
Tyring et al. [20], 2020	Pooled post hoc analysis of 2 RCTs	1640 patients with moderate-to-severe acne (EGSS 3–4)	Tretinoin 0.05% lotion vs. vehicle, once daily	12 weeks	Tretinoin showed statistically significant improvement over vehicle in all Acne-QoL domains: self-perception (7.4 vs. 6.7), role—emotional (6.8 vs. 6.0), role—social (4.8 vs. 4.6), and acne symptoms (6.5 vs. 5.6), all *p* < 0.05. Strong correlation between symptom improvement and QoL gain (r = 0.66–0.69). EGSS success (≥2-point improvement + clear/almost clear) achieved in 25.4% vs. 15.4% (*p* = 0.006). Safety profile favorable.
Babayeva et al. [21], 2011	Single-blind, RCT, comparative	46 adults (18–35 y) with mild-to-moderate facial AV; 23 per group	Tretinoin 0.05% + clindamycin vs. salicylic acid 3% + clindamycin	12 weeks	Both treatments significantly reduced total, inflammatory, and non-inflammatory lesion counts. Tretinoin + CDP led to faster reduction in total lesions (50% reduction at week 2 in 21.7% vs. 0%, *p* = 0.049) and greater improvement in inflammatory lesions at week 4 (*p* = 0.005). No differences at week 12. Mild-to-moderate side effects in both groups, mostly transient. Quality of life improved similarly in both arms.
Jarratt et al. [22], 2012	Randomized, double-blind, vehicle-controlled trial	1649 participants with acne vulgaris	Clindamycin 1.2% + tretinoin 0.025% (CT gel) vs. clindamycin alone, tretinoin alone, or vehicle	12 weeks	CT gel was significantly more effective than monotherapies and vehicle in reducing total lesions (vs. all), non-inflammatory lesions (vs. clindamycin), and inflammatory lesions (vs. tretinoin). More patients achieved ≥2-grade improvement in ISGA. Tolerability was comparable to tretinoin alone; most irritation occurred at week 2. No increase in adverse events with CT gel.
Kircik [23], 2009	Two RCTs (12-week, multicenter, double-blind)	147 patients with moderate-to-severe acne (12–46 years; 59.9% female)	BPO 5% + clindamycin 1% + RAM 0.04% (Group 1) vs. BPO 5% wash (AM) + CPT gel (clindamycin 1.2% + tretinoin 0.025%) (PM) (Group 2), both once daily	12 weeks	Mean reduction in inflammatory lesions: 48% (Group 1) vs. 42% (Group 2). At week 12, 80.8% in Group 1 and 87.8% in Group 2 were rated “clear” or “almost clear”. Greater % of patients achieved ≥75% reduction in inflammatory lesions with Group 1 (52.8% vs. 43.7%). Group 1 showed significantly faster improvement in inflammatory lesions at weeks 4 and 8. Group 2 had significantly less dryness and peeling at week 8. Pruritus absent in 91.7% vs. 79.4% (Group 2 vs. Group 1) at week 12. Both regimens were well tolerated.
Nilfroushzadeh et al. [24], 2009	Single-blind RCT	42 females (15–25 y) with mild-to-moderate acne (randomized 1:1:1)	Group A: 1% clindamycin lotion BID (C lotion) Group B: 1% clindamycin + 0.025% tretinoin QHS (CT lotion) Group C: 1% clindamycin + 2% salicylic acid lotion BID (CS lotion)	12 weeks	CS lotion showed significantly greater efficacy than clindamycin alone in reducing total lesion count (TLC, −81.80%) and acne severity index (ASI, −73.73%). CT lotion achieved −72.20% TLC and −55.95% ASI. Differences between CT and CS were not statistically significant. CS lotion also had best results for CCN (−87.05%) and PPN (−84.5%). No significant differences found for open comedones or pustules. Adverse events were mild; slight irritation in 21.4% (CT), burning in 50% (CS), none in clindamycin.
Pariser et al. [25], 2010	Randomized, investigator-blinded, phase 4 trial	247 patients (mean age: 18.5 years) with moderate facial acne (≥12 years)	Tretinoin 0.04% gel microsphere + BPO 5% wash, morning/morning vs. morning/evening regimen	12 weeks	Lesion count reduction similar between morning/morning and morning/evening regimens; no significant difference in tolerability.
Rosso et al. [26], 2023	Two phase 3, double-blind, vehicle-controlled RCTs	Study 1: 424 subjects (E-BPO/T n = 281; vehicle n = 143) Study 2: 434 subjects (E-BPO/T n = 290; vehicle n = 144) Subjects ≥9 years with moderate-to-severe facial acne (IGA 3–4)	E-BPO/T: 3% encapsulated benzoyl peroxide + 0.1% encapsulated tretinoin cream, QHS vs. vehicle cream QHS, both after cleansing	12 weeks	IGA success (clear/almost clear + ≥2-grade improvement): Study 1: 38.5% (E-BPO/T) vs. 11.5% (vehicle); Study 2: 25.4% vs. 14.7% (*p* < 0.05). Inflammatory lesion count: Study 1: −21.6 (E-BPO/T) vs. −14.8; Study 2: −16.2 vs. −14.1 (*p* < 0.05). Noninflammatory lesion count: Study 1: −28.7 vs. −19.8; Study 2: −24.2 vs. −17.4 (*p* < 0.001). Improvements seen from week 2. AEs mainly mild to moderate and cutaneous. Local tolerability scores comparable to vehicle.
Trifu et al. [27], 2011	RCT, double-blind, 3 arms	77 patients (age 18–45) with mild-to-moderate facial acne	CB-03-01 cream 1% QHS (n = 30) Tretinoin cream 0.05% QHS (n = 32) Placebo cream (vehicle) QHS (n = 15)—all applied once daily to affected areas for 8 weeks	8 weeks	CB-03-01 vs. placebo (week 8): TLC: −28.3% (*p* = 0.0017) ILC: −27.9% (*p* = 0.0134) ASI: −23.4% (*p* = 0.009) IGA success (score ≤1): 22% vs. 7% (no *p*-value reported) CB-03-01 vs. tretinoin: no statistically significant differences for any variable (*TLC*, *ILC*, *ASI*, or *IGA*). Irritation score significantly lower with CB-03-01 than tretinoin at week 1 (*p* = 0.0412); no serious AEs reported.
Zaenglein et al. [28], 2013	Phase IV, open-label single-arm study	97 patients (12–30 y) with moderate to severe acne (IGA 3–4, mean age 18.3 ± 4.2 y)	Oral minocycline ER 1 mg/kg QD + topical clindamycin phosphate 1.2%/tretinoin 0.025% QD + 6% BP foaming cloths QD	12 weeks	At week 12, 89% showed ≥1-grade IGA improvement and 55% ≥2-grade. Lesion counts reduced by 56.5% (total), 61.8% (inflammatory), 48.8% (non-inflammatory). AEs mild/moderate in 19%.
Zeichner et al. [29], 2013	Single-blind, randomized, controlled study	40 patients aged 12–53 years with mild to severe facial acne (80% female)	Tretinoin 0.025% + clindamycin 1.2% gel (TCP) once daily vs. TCP + BPO 6% cleansing cloths (evening)	12 weeks	PGA success (clear/almost clear) at week 12: 68.4% in TCP + BPO vs. 60.0% in TCP alone (*p* = NS). SSA success (clear/almost clear) at week 12: 26.3% vs. 10.0% (*p* = NS). No statistically significant difference in efficacy, but trend favoring TCP + BPO.

Abbreviations: BPO (benzoyl peroxide), CADI (Cardiff Acne Disability Index), CT gel (combination therapy gel: clindamycin 1.2% + tretinoin 0.025%), DLQI (Dermatology Life Quality Index), EGSS (Evaluator’s Global Severity Score), ISGA (Investigator’s Static Global Assessment), IGA (Investigator’s Global Assessment), IL (inflammatory lesions), NI (non-inflammatory lesions), QoL (quality of life), RCT (randomized controlled trial), SSA (Subject Self-Assessment), TCP (tretinoin 0.025% + clindamycin 1.2% gel), TLS (total lesion score), TLC (total lesion count), ASI (acne severity index), PPN (papules and pustules number), CCN (closed comedone number), mGAGS (modified Global Acne Grading System), IGE (Investigator’s Global Evaluation), RAM (retinoic acid microsphere), CPT (clindamycin phosphate and tretinoin).

Novel formulations such as tretinoin 0.05% lotion have been assessed in post hoc analyses and phase 3 RCTs. In two large RCTs (n = 1640), treatment success (≥2-point Evaluator’s Global Severity Score [EGSS] improvement plus clear/almost clear) was achieved in 38.5% and 25.4% of patients at week 12, respectively, compared to 11.5% and 14.7% in the vehicle groups (*p* < 0.05) [18,20]. Similar findings were reported in Hispanic (n = 766) [12] and Asian (n = 69) [17] populations, with improved quality-of-life (QoL) scores [20]. In a phase IV open-label study (n = 544), tretinoin 0.04% and 0.1% gel applied via pump led to moderate or greater improvement in 72% of subjects, and 25% were rated as clear or almost clear at week 12. Treatment compliance exceeded 95% [15]. In preadolescents (n = 110), another RCT confirmed a significant reduction in non-inflammatory lesions with tretinoin vs. vehicle (−19.9 vs. −9.7, *p* = 0.04). Investigator’s Global Assessment (IGA) success (≥2-point improvement) was achieved in 25.5% vs. 13.0% (*p* = 0.02), with mild application-site reactions [16]. In a split-face trial comparing encapsulated vs. non-encapsulated tretinoin, both achieved similar efficacy, though tolerability was superior with encapsulation [26].

Comparative trials with other active agents have also been conducted. In an RCT (n = 46), tretinoin 0.05% + clindamycin + salicylic acid showed greater lesion reduction and faster onset of action than salicylic acid + clindamycin alone (50% vs. 21.7% reduction at week 2; *p* = 0.049) [21].

Topical tretinoin has also been evaluated as part of combination regimens. In a large RCT (n = 1649), a clindamycin 1.2% + tretinoin 0.025% gel demonstrated superior reduction in lesion count and in Investigator’s Static Global Assessment (ISGA) scores compared to each monotherapy or vehicle [22]. In another comparative study (n = 750), the same combination achieved greater ISGA success (46%) vs. monotherapies (31% with tretinoin, 33% with clindamycin; *p* < 0.02) [13]. Additional studies comparing morning vs. evening application of tretinoin 0.04% microsphere + BPO 5% showed no difference in efficacy or tolerability [25]. Lastly, two phase 3 RCTs (n = 858 total) evaluating microencapsulated tretinoin 0.1% + encapsulated benzoyl peroxide (BPO) 3% confirmed superior IGA success vs. vehicle (38.5% and 25.4% vs. 11.5% and 14.7%, respectively; *p* < 0.05) [26]. The triple combination regimen (BPO/clindamycin/tretinoin) has also been explored in three RCTs (n = 97 and n = 40), revealing good clinical responses [23,28,29].

#### 3.1.2. Photoaging (Table 3)

A pivotal study by Bagatin et al. [30] compared 0.3% adapalene gel to 0.05% tretinoin cream over 24 weeks in 86 photoaged women. Both treatments led to significant improvement in clinical signs such as periorbital and forehead wrinkles, pigmentation and overall photodamage scores, with no significant differences in efficacy or tolerability. McDaniel et al. [31] evaluated a double-conjugated cream versus 0.025% tretinoin in a split-face study. Both treatments improved fine lines, dyschromia, and erythema after 12 weeks. Sumita et al. [8] conducted an evaluator-blinded trial comparing 0.05% tretinoin cream (applied three times weekly) with 5% tretinoin peel (every 2 weeks) on photoaged forearms of 24 postmenopausal women. Both regimens significantly reduced photoaging scores and actinic keratoses. The cream improved dermal echogenicity and increased Ki67 expression (an established nuclear marker of cell proliferation indicating active keratinocyte turnover), while the peel was more effective in stabilizing field cancerization. In a mechanistic study, Chien et al. [32] found that circulating levels of tretinoin precursors (retinol, retinaldehyde and retinyl esters) correlated with clinical response to tretinoin 0.02% over 24 weeks. Suppression of MMP2 gene expression was associated with improvement in fine wrinkles.

**Table 3:** Main studies about tretinoin for treatment of photoaging.

Study	Design	Population	Tretinoin Formulation	Comparator	Main Outcomes
Bagatin et al. [30], 2018	RCT, 24 weeks	86 women with facial photoaging	0.05% cream	0.3% adapalene gel	Comparable efficacy; improvement in wrinkles and pigmentation; similar tolerability.
McDaniel et al. [31], 2017	Split-face, 12 weeks	48 patients with photodamage	0.025% cream	Double-conjugated retinoid cream	Similar improvement; tretinoin associated with more irritation.
Sumita et al. [8], 2018	Evaluator-blinded RCT, 24 weeks	24 postmenopausal women (forearms)	0.05% cream (3×/week)	5% tretinoin peel (biweekly)	Both effective; cream improved dermal echogenicity and Ki67; peel more effective for field cancerization.
Chien et al. [32], 2022	Mechanistic study, 24 weeks	24 patients with moderate-to-severe photoaging	0.02% cream	Retinol + retinyl esters	Similar clinical improvement; tretinoin increased erythema; MMP2 suppression correlated with improvement in fine wrinkles.
Siddiqui et al. [33], 2024	Systematic review	Multiple RCTs reviewed	Various (0.02–0.1%)	Retinol, glycolic acid, others	Tretinoin superior in efficacy; AEs (irritation, erythema) more frequent at higher concentrations.
Kligman and Draelos [34], 2004	Open-label trial	32 patients with facial photoaging	0.25% cream	None (titration protocol)	High-strength tretinoin tolerated with gradual escalation and moisturizers; early improvement observed.

Notes: MMP2: matrix metallo-proteinase 2; RCT: randomized clinical trial; AEs: adverse events.

Finally, a systematic review by Siddiqui et al. [33], including 25 studies, concluded that tretinoin was consistently effective in improving both clinical and histological signs of photoaging. Comparisons with other topical agents, such as retinol, glycolic acid, and antioxidants, indicated the superior efficacy of tretinoin across most outcome measures.

### 3.2. Off-Label Use

#### 3.2.1. Melasma and Post-Inflammatory Hyperpigmentation

Faghihi et al. [35] compared 1% tretinoin peel to 70% glycolic acid in 63 women with melasma, reporting similar reductions in the Melasma Area Severity Index (MASI) but better tolerability with tretinoin. The fixed triple combination of fluocinolone acetonide 0.01%, hydroquinone 4%, and tretinoin 0.05% has consistently demonstrated superior efficacy compared to its individual components or alternative treatments. In an RCT including 260 women with moderate-to-severe melasma, Chan et al. [36] reported significantly greater MASI score reduction at week 8 in the triple combination group compared to hydroquinone alone or vehicle, with 77% of patients rated as markedly improved or clear. Similar results were confirmed by Gong et al. [37] in a Chinese population. The benefit of the triple combination was also observed in Grimes and Watson’s study [38], in which the addition of tretinoin 0.025% to a hydroquinone-based regimen resulted in progressive improvement in MASI scores over 12 weeks. Hu et al. [39] compared a generic and a reference triple combination in 60 Chinese patients and found both to be equally effective and well tolerated, with no significant differences in clinical response or AEs. A systematic review and meta-analysis by Pennitz et al. [40], including 18 randomized controlled trials, confirmed that the triple combination consistently outperformed monotherapies and ranked among the most effective self-applied treatments for melasma. However, the same meta-analysis also recommended topical tretinoin monotherapy in selected patients, including for long-term maintenance [40], a recommendation that is similarly applicable to long-term post-inflammatory hyperpigmentation management [41].

The efficacy of topical tretinoin has also been compared to physical therapies. Kroon et al. [42] evaluated a fractional 1550 nm laser treatment versus a triple combination in 40 patients, showing comparable reductions in MASI after 12 weeks (−4.9 vs. −4.4, respectively). Leerapongnan et al. [43] found that an erbium fiber laser achieved greater MASI reduction than tretinoin 0.05% cream (−2.58 vs. −1.50; *p* = 0.001), though both groups showed significant improvement. Laser-assisted delivery has also been explored. Woodhall et al. [44] demonstrated that the adjunctive use of a hydroquinone/tretinoin regimen with IPL significantly improved hyperpigmentation and skin laxity scores compared to IPL with a placebo (≥75% overall improvement in 72% vs. 19% of patients). Schlessinger et al. [45] observed a 27.2% reduction in hyperpigmentation scores when tretinoin and hydroquinone were applied after IPL.

#### 3.2.2. Acanthosis Nigricans

A recent systematic review [7] found six RCTs (172 patients in total) evaluating topical tretinoin for the treatment of *acanthosis nigricans* (AN). Tretinoin 0.025% and 0.05% were similarly effective in reducing pigmentation [46]. Tretinoin 0.025% was more effective than 10% urea cream [47] and 15% trichloroacetic acid [48] and similar to 0.1% adapalene [49]. Tretinoin 0.05% was comparable to a fractional 1550 nm erbium laser in decreasing pigmentation, although the laser was superior in improving cutaneous texture [43]. The authors concluded that topical tretinoin merited a grade A recommendation, based on three level 1b studies and three level 2b studies [7]. A recent RCT (not included in the previous review) assessed the effectiveness of tretinoin 0.05% versus glycolic acid peeling 70%. Tretinoin showed better clinical responses for AN in the axilla region [50].

#### 3.2.3. Rosacea

In an RCT by Chang et al. [51], the topical combination of clindamycin phosphate 1.2% and tretinoin 0.025% gel was evaluated for the treatment of rosacea. The study found no significant difference between the treatment and placebo groups in physician-assessed global improvement, quality of life, or lesion count. Another RCT by Freeman et al. [52] with 30 patients compared the same combination with placebo and described a significant improvement in papulo-pustular rosacea.

#### 3.2.4. Striae Distensae (Stretch Marks)

*Striae distensae* (SD) is a very frequent condition. Initially, SD can present as linear erythematous/violaceous lesions (*Striae rubra*) with no marked skin depression. Over time, SD become white and atrophic (*Striae albae*). A systematic review and meta-analysis from 2020 that included 14 RCTs (651 patients) revealed that the most effective intervention (when evaluating clinical effectiveness and patient satisfaction) was radiofrequency combined with topical tretinoin [53]. A systematic review from 2016 found four RCTs on tretinoin for the treatment of SD. These studies revealed that tretinoin 0.05%/0.1% was effective in the treatment of *Striae rubrae*, albeit of limited value in *Striae albae* [54]. A clinical trial (n = 30) evaluated the efficacy of intralesional injection of platelet-rich plasma (PRP) versus topical tretinoin 0.05% in the treatment of SD. Both treatments improved skin appearance and increased collagen and elastic fibers in the dermis. The improvement was more marked in the SD under PRP [55]. Another RCT (n = 32) compared tretinoin 0.05% versus dermabrasion for the treatment of *Striae rubra*. Both treatments had similar efficacy [56].

#### 3.2.5. Flat Warts (Verruca Plana)

A prospective comparative study (n = 100) [5] assessed the effectiveness of trichloroacetic acid (TCA) 30%, tretinoin 0.05%, and 5-FU 5% for the treatment of plane warts. At week 12, all the therapies had decreased the number of warts. The lesion numbers had reduced to 14%, 52%, and 27%, respectively. TCA was the most effective treatment, although it was associated with hypo- or hyperpigmentation in 25% of patients (versus 6% with tretinoin and 9% with 5-FU). In an RCT (n = 60) [57], tretinoin led to complete clearance of flat warts in 53% of cases and TCA in 90%. AEs were not reported in this study.

#### 3.2.6. Disorders of Keratinization

Topical tretinoin is often employed in clinical practice for various keratinization disorders such as *Keratosis pilaris*, Darier disease, granular parakeratosis, and lamellar ichthyosis. However, the existing evidence is limited to case reports and small, uncontrolled series; no RCTs or robust clinical studies have been identified to date, and its use remains largely empirical.

#### 3.2.7. Prevention of Hypertrophic Scars and Keloids

An RCT [4] (26 patients, 44 wounds) assessed the application of topical silicone gel, tretinoin 0.025%, and a placebo over 24 weeks on postoperative wounds for the prevention of hypertrophic scars or keloids. The subjects applied a topical treatment twice daily after their stitches were removed. Both agents effectively prevented hypertrophic scars and keloids and improved scar appearance compared with the control. No significant difference was observed between silicone gel and tretinoin. Another RCT [58] evaluated the efficacy of using hydroquinone 4% plus tretinoin 0.05% compared to the standard treatment to enhance aesthetic outcomes resulting from electrodesiccation and curettage therapy for superficial truncal basal cell carcinomas. Both treatments were applied 3 weeks prior to and 3 weeks after the procedure (post-therapy beginning after >75 percent reepithelialization). All of the expert graders described a higher incidence of treatment success (excellent or good wound appearance) with hydroquinone/tretinoin therapy.

#### 3.2.8. Treatment of Hypertrophic Scars/Post-Burn Scars

A prospective study (n = 15) [59] utilizing 0.05% tretinoin for one year on facial post-burn scarring revealed a significant reduction in skin resistance and elastance (determined by biomechanical analysis), indicating improved distensibility. Another prospective comparative study analyzed 77 patients, ages 6 to 46 years, with perioral burn sequelae. The use of tretinoin (0.01–0.05%) and glycolic acid (5–7%) over a 3-month period led to a significant improvement in mouth-opening measurements [60].

#### 3.2.9. Androgenetic Alopecia

An RCT (n = 31) compared the efficacy of 5% minoxidil and 0.01% tretinoin once daily versus 5% minoxidil applied twice daily in the treatment of androgenetic alopecia. There were no significant differences between the two treatment groups in macrophotographic variables such as changes in total hair count, non-vellus hair count, anagen hair ratio, and mean hair diameter [61].

Sulfotransferase enzymes in the hair follicle transform minoxidil into its active metabolite. A test measuring follicular sulfotransferase enzymatic activity showed an accuracy of 98% in identifying nonresponders to minoxidil [62]. In a prospective study (N = 20) [63], five applications of topical tretinoin 0.1% increased follicular sulfotransferase expression in 43% of patients initially predicted to be non-responders. These subjects were then converted into responders to minoxidil.

#### 3.2.10. Alopecia Areata

A prospective comparative study on 80 patients with limited alopecia areata divided them into four groups: topical steroids, tretinoin 0.05%, dithranol 0.25%, and petrolatum jelly. Treatments were applied for 3 months. Good regrowth was observed in 70%, 55%, 35%, and 20%, respectively. Topical corticoids and tretinoin were the most effective therapies [64].

#### 3.2.11. Wound Healing

An RCT [65] including 24 patients with diabetic foot ulcers compared 4 weeks of daily short-contact treatment (10 min per application) with tretinoin 0.05% solution versus placebo. At week 16, 46% of ulcers in the tretinoin group had healed completely, versus 18% of ulcers in the control group. Short-contact tretinoin therapy significantly reduced ulcer area (*p* < 0.01) and depth (*p* = 0.02) compared with placebo. No necrotic tissue, nor significant erythema or edema, was observed in the tretinoin group. 

#### 3.2.12. Prevention and Treatment of Skin Cancer

##### Treatment of Actinic Keratosis

Treatment of AK can be challenging. Field-directed therapies can be poorly tolerated, and this may compromise adherence. An RCT (n = 61) compared tretinoin 0.05% versus oral isotretinoin 10 mg/day for 6 months for the treatment of actinic keratosis (AK) on the face and forearms. Both agents significantly decreased the number of AKs (around 28%), stratum corneum thickness and expression of Bax (a protein linked to apoptosis) and p53. AEs were mild and well tolerated [66]. Another RCT (intrasubject, n = 24) evaluated tretinoin 0.05% for 24 weeks versus tretinoin 5% peel for the treatment of field cancerization on the forearms. Both treatments reduced AK count by 60% with no difference between them [8]. Also, a significant reduction in photoaging signs was observed.

##### Prevention of Keratinocyte Carcinoma

A Veterans Affairs RCT (n = 1131) that evaluated high-dose topical tretinoin (0.1% cream) for 1.5–5.5 years for keratinocyte carcinoma prevention showed no differences between intervention and control groups in the rate of BCC or invasive SCC at 5 years [67]. However, a retrospective study (n = 327) [68] showed that the combination of imiquimod 5% cream, 5-fluorouracil 2% solution, and tretinoin 0.1% cream (30 applications over 42 nights) indicated as a field treatment was effective at reducing the incidence of new keratinocyte carcinomas (KCs) for at least one year (OR = 0.06, 95% CI: [0.02, 0.15]).

##### Treatment of Non-Melanoma Skin Cancer

A retrospective study (186 carcinomas in 133 patients) [69] evaluated diverse topical treatment regimens for the treatment of KC: 30 applications of one of three combinations (imiquimod 5%/tretinoin 0.1%, 5-fluorouracil 2%/tretinoin 0.1%, or imiquimod 5%/5-fluorouracil 2%/tretinoin 0.1%) plus cryotherapy every 2 weeks. The imiquimod/5-fluorouracil/tretinoin combination was the most effective treatment regimen, with clearance rates of 100% (30/30) for superficial and nodular basal cell carcinoma (BCC); 67% (2/3) for morpheaform BCC; 100% (17/17) for in situ squamous cell carcinoma (SCC); and 100% (18/18) for invasive SCC. A single recurrence was observed during a 3-year follow-up (morpheaform BCC). The patients under this combination were instructed to apply one-fifth of a packet of imiquimod 5%, 1 drop of 5-FU 2%, and one-fifth of a pea-sized quantity of tretinoin 0.1% to a bandage and then apply it to the tumor overnight, five times a week for 6 weeks (30 applications over 42 days). In a case series, 13 patients with KC (superficial, nodular, and morpheaform BCC; in situ and invasive SCC) used the same topical combination therapy with complete clearance of all tumors [70].

#### 3.2.13. Tretinoin Prior to Energy-Based Therapy

An RCT of 36 subjects with moderate/severe wrinkling around the eyes and lips assessed the use of 4% hydroquinone/0.05% tretinoin or placebo for 90 days. All individuals underwent intense pulsed light treatment (at days 30 and 60). At day 90, ≥75% overall improvement was reported in 72% and 19% of subjects in the intervention group (+IPL) and the placebo (+ IPL) group, respectively. Hydroquinone/tretinoin was associated with significantly lower hyperpigmentation, telangiectasia, and laxity scores at day 90 (*p* ≤ 0.05) [44]. In a prospective study [71], 18 Asian patients received tretinoin gel 0.1% and hydroquinone 5% for 6 weeks and then underwent Q-switched ruby laser therapy to treat periorbital skin hyperpigmentation. Excellent clearing was reported in 39% of cases and a good response in 44%. Post-inflammatory hyperpigmentation was reported in two patients. Similar results were observed in another prospective study (n = 19) using 0.1% tretinoin gel and 5% hydroquinone ointment containing 7% lactic acid [72].

In a review of the literature from 2016, the authors recommended the use of 0.1% tretinoin cream for 3 months prior to ablative lasers, discontinuing one day before beginning treatment. For non-ablative laser resurfacing, the tretinoin dose should be reduced to 0.05% [73]. However, the authors acknowledged that these recommendations were mainly based on anecdotal evidence and personal experience.

#### 3.2.14. Tretinoin Prior to Botulinum Toxin

In an RCT [45], 61 subjects who underwent upper facial therapy with botulinum toxin Type A were randomly allocated to treatment with 4% hydroquinone and 0.05% tretinoin cream (plus skin care regimen) or a standard skin care regimen for 120 days. Hydroquinone/tretinoin cream significantly reduced hyperpigmentation and milder fine lines/wrinkles and led to significantly superior overall ratings for each of nine patient assessments (including skin tone, facial texture, and facial overall improvement) at days 30, 90 and 120 (*p* ≤ 0.05).

## 4. Safety and Tolerability of Topical Tretinoin

Tretinoin presents a good safety profile. We did not find severe AEs in the studies included in this review. Most AEs were local reactions (such as increased photosensitivity) and were mild to moderate, and no long-term complications were reported. Regarding systemic AEs, percutaneous absorption of topical tretinoin is minimal (1–2%), even after repeated application [74]. A systematic review from 2011 that included 14 studies on topical tretinoin did not find clear evidence of an association between the topical use of tretinoin and the development of noncutaneous adverse events [75].

While it is advisable not to use topical tretinoin during pregnancy, three prospective studies (n = 343) [76,77,78] and one retrospective study (n = 215) [79] found no association between exposure to tretinoin during pregnancy and increased risk of congenital malformations.

## 5. Discussion

Despite decades of use, topical tretinoin therapy remains dynamic, with ongoing research exploring new therapeutic applications and the development of innovative formulations to improve tolerability and adherence. Tretinoin has been shown to be a well-tolerated and suitable alternative for a wide range of dermatological conditions (Table 4), especially for *acne vulgaris* and photoaging, where its usefulness is supported by extensive clinical trial data [10,14,19,22,30,31]. Tretinoin is considered a first-line treatment for mild-to-moderate acne, including inflammatory and non-inflammatory lesions [80,81]. Furthermore, tretinoin has shown efficacy and safety across diverse phototypes, including patients with skin of color [17]. Topical retinoids are also crucial for maintenance therapy, helping to reduce antibiotic use, scarring, and post-inflammatory hyperpigmentation (PIH) [6]. Moreover, tretinoin is the gold standard treatment for preventing and reversing signs of cutaneous aging [33] due to its ability to modulate epidermal differentiation, stimulate collagen synthesis, and promote dermal remodeling [8,31]. Compelling evidence reveals improvement in fine and coarse wrinkles, mottled pigmentation, uneven skin tone and rough cutaneous texture [33]. Furthermore, tretinoin could enhance clinical responses after energy-based or in-office procedures used to treat photoaging [44,71,72,73], although direct clinical evidence from these procedures is limited and is not standardized in protocols.

Regarding off-label indications, multiple RCTs have shown the efficacy of tretinoin in reducing pigmentation in AN [7] and improving *Striae distensae* [53,54], especially active lesions (*Striae rubrae*), conditions that can significantly impact quality of life. Tretinoin has proven to be superior to other frequently used agents such as 10% urea cream, TCA, and glycolic acid in AN [48,50]. For melasma and PIH, while tretinoin monotherapy has demonstrated efficacy [35], combination therapies frequently yielded superior results: triple combination and tretinoin/hydroquinone have been evaluated in multiple RCTs [40] showing significant reduction in pigmentation. This suggests that for complex pigmentary disorders, a multi-modal approach targeting diverse pathogenic pathways is often optimal. Topical tretinoin can also be a good alternative not only to treat photoaging, but for the control and stabilization of field cancerization, and could be used as a maintenance therapy after treatment with field-directed therapies such as topical 5-fluorouracil.

The most frequently reported AEs of topical tretinoin include itching, scaling, erythema, and dryness. These reactions are typically mild to moderate in severity and transient [82]. Strategies to mitigate irritation include introducing therapy with lower concentrations (standard concentrations range from 0.025% to 0.1%), applying the product two or three nights/week initially, and the use of moisturizers, together with broad-spectrum sunscreens (SPF 30 or higher) (Table 5). Effective patient counseling and proactive side effect management are essential for treatment adherence. Emphasizing the necessity of consistent, long-term use, particularly for chronic conditions, is key.

## 6. Limitations

Our work has several limitations. It is not a systematic review or a meta-analysis. We have found, in general, low-level evidence for conditions different from acne and photoaging. There is a recognized need for large-scale, high-quality RCTs, with standardized outcome measures, to establish clearer clinical guidelines and optimized protocols. 

## 7. Conclusions

Topical tretinoin impacts cutaneous cellular proliferation, differentiation, inflammation, skin pigmentation, and extracellular matrix remodeling. Multiple clinical trials show its efficacy in the management of acne, photoaging, AN, and *striae distensae*. Tretinoin has also been successfully indicated in diverse dermatological conditions, frequently enhanced by combination therapies, including pigmentary disorders, flat warts, and scarring, and could be indicated as a pretreatment prior to energy-based therapies or in-office procedures.

## Figures and Tables

**Table 1:** Mechanism of action of topical tretinoin.

Mechanism	Associated Clinical Effect
Keratolytic activity	Promotes desquamation and prevents microcomedone formation
Regulation of keratinocyte proliferation and differentiation	Normalizes epidermal turnover; beneficial in acne, psoriasis and keratinization disorders
Fibroblast activation	Stimulates dermal repair and collagen production
Induction of collagen synthesis and recycling	Improves skin texture and elasticity; useful in photoaging and scarring
Inhibition of matrix metalloproteinases (MMP-1 and MMP-8)	Prevents collagen degradation
Reduction of epidermal melanin (via tyrosinase inhibition)	Helps lighten hyperpigmentation such as melasma and post-inflammatory hyperpigmentation
Activation of nuclear retinoic acid receptors (RARα, RARβ, RARγ)	Regulates gene transcription related to differentiation, inflammation and tissue remodeling
Preferential interaction with RARγ	Highly expressed in epidermis; key in therapeutic response and cutaneous irritation
Suppression of inflammatory mediators (e.g., AP-1 inhibition)	Contributes to anti-inflammatory effects in conditions like acne and rosacea
Upregulation of CRABP-II expression in the skin	Enhances intracellular transport and bioavailability; modulates treatment intensity

**Table 4:** Dermatologic conditions where topical tretinoin has shown good clinical response.

Skin Aging	Photoaging [33]
Inflammatory disorders	*Acne vulgaris* [15]
Pigmentary disorders	Melasma [37] Post-inflammatory hyperpigmentation [41] *Acanthosis nigricans* [7]
Scarring	*Striae distensae* [53] Prevention of hypertrophic scars/keloids [4] Treatment of post-burn scars [60]
Infections	Flat/plane warts [5]
Disorders of keratinization	Granular parakeratosis Darier’s disease *Keratosis pilaris*
Alopecia	Alopecia areata [64] Androgenetic alopecia [61]
Skin neoplasms	Treatment of AK and field cancerization [66] Prevention and treatment of keratinocyte carcinoma * [67]

Abbreviations: AK, actinic keratosis. * Combination of imiquimod 5% cream, 5-fluorouracil 2% solution, and tretinoin 0.1% cream (30 applications over 42 nights).

**Table 5:** Strategies to mitigate irritation when using topical tretinoin [80].

Start Topical Tretinoin Therapy with Lower Concentrations (0.025% or 0.05%)
Apply the product two or three nights/week initially, and gradually increase the frequency according to tolerability.
Avoid excessive skin cleansing.
Wait 20–30 min after cleansing to ensure skin is completely dry before application.
Avoid aggressive exfoliation of the skin.
Intensive use of emollients and moisturizers.
Recommend photoprotection, including broad-spectrum sunscreens (SPF 30 or higher).
